# Effects of Social Media Use for Health Information on COVID-19–Related Risk Perceptions and Mental Health During Pregnancy: Web-Based Survey

**DOI:** 10.2196/28183

**Published:** 2022-01-13

**Authors:** Qian Wang, Luyao Xie, Bo Song, Jiangli Di, Linhong Wang, Phoenix Kit-Han Mo

**Affiliations:** 1 National Center for Women and Children's Health Chinese Center for Disease Control and Prevention Beijing City China; 2 Center for Health Behaviours Research, Faculty of Medicine School of Public Health and Primary Care The Chinese University of Hong Kong, Prince of Wales Hospital Hong Kong Hong Kong; 3 National Center for Chronic and Noncommunicable Disease Control and Prevention Chinese Center for Disease Control and Prevention Beijing City China

**Keywords:** COVID-19, pregnant, social media use, risk perception, worry, depression

## Abstract

**Background:**

Social media has become an important source of health information during the COVID-19 pandemic. Very little is known about the potential mental impact of social media use on pregnant women.

**Objective:**

This study aims to examine the association between using social media for health information and risk perception for COVID-19, worry due to COVID-19, and depression among pregnant women in China.

**Methods:**

A total of 4580 pregnant women were recruited from various provinces of China. The participants completed a cross-sectional, web-based survey in March 2020.

**Results:**

More than one-third (1794/4580, 39.2%) of the participants reported always using social media for obtaining health information. Results of structural equation modeling showed that the frequency of social media use for health information was positively associated with perceived susceptibility (*β*=.05; *P*<.001) and perceived severity (*β*=.12; *P*<.001) of COVID-19, which, in turn, were positively associated with worry due to COVID-19 (*β*=.19 and *β*=.72, respectively; *P<*.001). Perceived susceptibility (*β*=.09; *P*<.001), perceived severity (*β*=.08; *P*<.001), and worry due to COVID-19 (*β*=.15; *P*<.001) all had a positive association with depression. Bootstrapping analysis showed that the indirect effects of frequency of social media use for health information on both worry due to COVID-19 (*β*=.09, 95% CI 0.07-0.12) and depression (*β*=.05, 95% CI 0.02-0.07) were statistically significant.

**Conclusions:**

This study provides empirical evidence on how social media use for health information might have a negative impact on the mental health of pregnant women. Interventions are needed to equip this population with the skills to use social media properly and with caution.

## Introduction

COVID-19 is an infectious disease caused by a newly discovered coronavirus, named severe acute respiratory syndrome coronavirus 2 (SARS-CoV-2). The virus is known to have originated in Wuhan, China, in December 2019, and since then, it has spread rapidly, resulting in a global pandemic [1]. The rapid transmission of COVID-19 has caused massive disruption worldwide. As of February 14, 2021, more than 108 million people across 235 countries were infected with COVID-19, and more than 2 million associated deaths were reported [2].

Pregnant women are more susceptible to the morbidity and mortality associated with COVID-19, owing to the physiological changes that occur in the immune and cardiopulmonary systems during pregnancy [3,4]. A systematic review of 27 studies reported that 9.3% of pregnant women with COVID-19 were admitted to the intensive care unit, and 5.4% of them required mechanical ventilation [5]. As a uniquely vulnerable group, pregnant women require special attention and care during a pandemic. However, reduced access to health facilities during the COVID-19 pandemic caused significant psychological toll among people [6]. Pregnant women also experience serious stress and anxiety due to fear of infection, antenatal care suspension, boredom, frustration, and worries about the health of the fetus [7,8]. This may also lead to adverse effects for the child, such as inefficient mother-infant bonding [9] and the risk of inherited psychiatric illness [10]. Previous studies on pregnant women during the severe acute respiratory syndrome (SARS) outbreak in Hong Kong have suggested that 12.3% scored higher than the cut-off for depression, and 87.8% of pregnant women reported higher than moderate level of anxiety during the COVID-19 pandemic [11,12]. A recent study among 900 pregnant women in Canada found that, compared to the pre–COVID-19 period, pregnant women showed a significantly higher level of depression (from 15% to 41%) and anxiety (from 29% to 72%) during the COVID-19 pandemic [13]. Other studies have also shown that pregnant women experienced greater psychological distress than the general population during the pandemic [14,15].

In recent years, the widespread use of the internet has allowed individuals to access health information and receive support in their health care [16]. Women tend to be more involved in seeking health information on the internet [17], and web searches for health information have been found to be popular among pregnant women. For example, a study among 332 Chinese pregnant women showed that 88.7% of them used the internet to obtain health information, starting from the beginning of their pregnancies [18]. In general, between 28% and 95% of pregnant women use the internet for health information [19]. Common web-based search topics for obtaining health information included fetal development [18,20], stages of childbirth [20], antenatal pregnancy complications [21], and pregnancy nutrition [18]. The ease and accessibility of searching the internet during pregnancy met novice mothers’ information needs [21-23] and provided them with opportunities to share similar experiences and apprehensions with other women [23,24].

During the COVID-19 pandemic, the demand for information about the pandemic soared, and all published reports elevated public concerns about the serious threats of the pandemic. Social isolation measures resulted in consideration of the internet and social media as the primary sources of information about the pandemic [25,26]. Although social media has served as a powerful tool for disseminating health information, challenges and concerns over social media use have been raised. For example, there are serious concerns about misinformation and unsubstantiated rumors that were rapidly spread through social media, causing distrust and posing additional challenges for public health efforts to combat the pandemic [27]. Second, social media tends to overemphasize risks; repeated exposure to such platforms may, therefore, increase negative emotions such as panic and fear [28]. Third, compared to traditional media, social media not only provides information but also allows personal sharing and emotional expressions. Negative emotions are more likely to be conveyed on social media during an infectious disease pandemic [29]. A recent content analysis of messages posted to social media platforms in China during the COVID-19 period showed that personal posts are likely to attribute blame to other individuals or the government and express concerns about the pandemic [30]. Some studies have shown that social media exposure was positively related to increased anxiety, fear, posttraumatic stress disorder, and forming risk perceptions during previous outbreaks, such as the Middle East respiratory syndrome (MERS) outbreak, and the current COVID-19 crisis [29,31,32]. A study among factory workers in Shenzhen, China, conducted at the beginning of the COVID-19 pandemic, also found that higher exposure via unofficial web-based media was associated with higher depressive symptoms [25].

There could be more than a direct association between exposure to health information and mental distress during the COVID-19 period. Exposure to distressing health information on social media may intensify risk perceptions, leading to poor mental outcomes. Risk perception refers to an individual’s subjective evaluation of the possibility of a negative event; it consists of two key components: perceived susceptibility (ie, perception of the likelihood of contracting the disease) and perceived severity (ie, perception of the extent of harm of the disease). The cognitive model suggested that negative perceptions about a disease could increase worry or anxiety of one’s health status [33]. Evidence from previous public health crises (ie, Ebola and H1N1 outbreaks) also revealed that when a community crisis is repeatedly exposed in the media, considerable information about the risk of the health crisis could unintendedly lead to heightened anxiety and stress reactions [34,35].

Furthermore, information about the pandemic might change an individual’s perceived susceptibility and perceived harm of the disease [36,37]. Studies have found that during a global pandemic, mass media information would likely affect the perceived threat from the disease [38]; perceived threat, in turn, has shown to have a direct positive effect on negative mental outcomes, such as sadness, depression, anxiety, and anger [39]. Gender-based difference was also observed, with women perceiving higher levels of threat than men [38]. For pregnant women, pregnancy itself is characterized with heightened worries. Given the stress and uncertainty brought by the COVID-19 pandemic, using social media to obtain health information can be accompanied by various stressors, such as excessive information, long-term confinement, and fear of infection, all of which might increase the risk perceptions of this population [6].

Perception of susceptibility and severity may also lead to negative emotions, which in turn, could affect an individual’s mental health. The Appraisal Theory posits that emotions result from an individual’s evaluation or appraisal of an event, even in the absence of physiological arousal [40]. The appraisal process involves evaluation of two aspects of a situation: motivational relevance and motivational congruence. Motivational relevance assesses the relevance of the situation to one’s well-being, whereas motivational congruence evaluates the congruence of the situation with one’s goal. More intense emotional responses occur when a situation is judged to be highly relevant to one’s well-being and inconsistent to one’s goal [41]. It is therefore contended that the perception that one is at risk for COVID-19 infection and that the disease would have severe negative consequences will elicit negative emotions, leading to an adverse mental response. The association between risk perception of a disease and negative emotional reactions has been widely demonstrated in the literature [42,43]. In the context of COVID-19, studies from some Asian countries, including the Philippines and Vietnam, also support the findings that perceived susceptibility and impact of COVID-19 are related to negative emotions and poor mental health [44,45].

Based on the Appraisal Theory, this study aims to investigate whether and how social media use for health information might be associated with mental health outcomes among Chinese pregnant women during the COVID-19 era. In particular, the relationship between the use of social media for health information, risk perception (ie, perceived susceptibility and perceived severity of COVID-19), worry due to COVID-19, and depression were examined. It was hypothesized that using social media for health information would be associated with a higher level of risk perception that, in turn, would be associated with higher levels of worry and depression. Worry due to COVID-19 would also be positively associated with depression.

## Methods

### Study Design

A web-based, cross-sectional survey was conducted in March 2020. Pregnant women who were availing health services from maternal health care centers in Mainland China and who intended to continue the pregnancy were included in this study. Those who planned to terminate their pregnancy were excluded from the sample.

### Procedures

Participants were recruited from maternal health care centers of various provinces of China (ie, Beijing, Chongqing, Guangdong, Guangxi, Hainan, Shandong, Tianjin, and Xinjiang). Eligible women were first identified from medical records obtained from the center, and they were invited to participate in the survey through WeChat. Interested participants could access the web-based survey by scanning the quick response (QR) code or by clicking the link provided in the WeChat invitation message. Information about the purpose and procedure of the survey was provided on the first page of the web-based survey. Participants were assured about the confidentiality of the study and that refusal to participate in the survey would not affect any future services they would avail at the center. Informed consent was obtained from the participants by asking them to click on the “I agree” button on the first page of the survey. Ethical approval was obtained from the authors’ institution. A total of 4580 complete responses were collected (70% response rate).

### Measures

#### Sociodemographic Characteristics

Sociodemographic and pregnancy-related characteristics, including age, education level, parity, gestational age, and whether the participants had any pregnancy-related complications were collected.

#### Frequency of Social Media Use for Obtaining Health Information

Participants were asked to rate a single item about their frequency of using social media to seek health information in the past week. Responses are rated on a 4-point Likert scale ranging from 1 (never) to 4 (always).

#### Perceived Susceptibility to COVID-19

Participants were asked to rate 2 items on the extent to which they perceived that they and their family members would likely contract COVID-19. Responses were recorded on a 4-point Likert scale ranging from 1 (very little) to 4 (very much). A higher score indicated a higher level of perceived susceptibility. The internal reliability of the items was satisfactory (Cronbach α=.93).

#### Perceived Severity of COVID-19

Participants were asked to rate 3 items on their perceived consequences of COVID-19 (eg, “maternal infection with COVID-19 will affect the health of the newborn”). Items were rated on a 4-point Likert scale ranging from 1 (very little) to 4 (very much), with a higher score indicating a higher level of perceived severity. The internal reliability of the items was satisfactory (Cronbach α=.92).

#### Worry Due to COVID-19

Participants were asked to rate 4 items assessing their level of worry on various aspects related to COVID-19 (eg, “you will be infected with COVID-19 when you attend the prenatal check-up”). Items were rated on a 4-point Likert scale ranging from 1 (very little) to 4 (very much), with a higher score indicating a higher level of worry. The internal reliability of the items was satisfactory (Cronbach α=.91).

#### Depression

Depression was measured using the Patient Health Questionnaire-9 [46], which has been validated and used in the Chinese population [47,48]. Participants were asked to rate how often they have been bothered by COVID-19–related symptoms in the past 2 weeks on a 4-point Likert scale ranging from 0 (not at all) to 3 (almost every day). Total scores ranged from 0 to 27, with a higher score indicating higher level of depression. A score of 0 to 4, 5 to 9, 10 to 14, 15 to 19, and 20 to 27 represented minimal, mild, moderate, moderately severe, and severe depression, respectively.

### Data Analyses

Descriptive statistics and zero-order correlations among all variables were performed. To evaluate the association between social media use for health information, risk perception, worry due to COVID-19 and depression, confirmatory factor analysis was conducted to assess the goodness of fit of the measurement model [49]. Structural equation modeling (SEM) was then performed to assess the hypothesized associations between the variables. Bootstrapping analysis, based on 2000 samples, was used to test the indirect effect. To evaluate the overall model fit, we considered the following indices: *χ*^2^ statistic, comparative fit index (CFI), incremental fit index (IFI), and root mean square error of approximation (RMSEA). Analyses were performed using AMOS 26 (IBM Corp) and tested using the maximum likelihood method.

## Results

### Descriptive Statistics of Study Participants

Of the 4580 participants, one-third (n=1538, 33.6%) were above 30 years of age; half (n=2334, 51%) had received postsecondary level of education; and a similar number (n=2300, 50.2%) were nulliparous. Slightly less than half (2143/4580, 46.8%) the participants were in their third trimester of pregnancy. A small number (n=310, 6.8%) of all participants reported having some pregnancy-related complications. Slightly less than half (n=2226, 48.6%) scored higher than the cut-off score for mild depression, and more than one-third (n=1794, 39.2%) reported always using social media for health information in the past week. More than one-third (n=2887, 63.1% to n=3104, 67.7%) of all participants showed a high level of susceptibility toward COVID-19. Furthermore, between 79.2% (n=3630) and 86.4% (n=3959) and between 68.5% (n=3136) and 75.5% (n=3462) of the 4580 participants reported a high level of severity and worry about COVID-19 (Table 1).

**Table 1:** Background characteristics of study participants (N=4580)

Characteristic		Value, n (%)	
**Age (years)**			
	≤19	62 (1.4)	
	20-25	967 (21.1)	
	26-30	2013 (44)	
	31-35	1197 (26.1)	
	36-40	274 (6)	
	≥41	67 (1.5)	
**Education level**			
	Primary or lower	117 (2.6)	
	Junior secondary	1130 (24.7)	
	Senior secondary	999 (21.8)	
	Matriculation	1218 (26.6)	
	Undergraduate	987 (21.6)	
	Postgraduate or higher	129 (2.8)	
**Parity**			
	Nulliparous	2300 (50.2)	
	Primiparous	2001 (43.7)	
	Multiparous	279 (6.1)	
**Gestational age**			
	First trimester (≤12 weeks)	904 (19.7)	
	Second trimester (13-26 weeks)	1533 (33.5)	
	Third trimester (≥27 weeks)	2143 (46.8)	
**Pregnancy-related complications**			
	No	4270 (93.2)	
	Yes	310 (6.8)	
**Depression (measured by PHQ-9^a^)**			
	Minimal (0-4)	2354 (51.4)	
	Mild (5-9)	1302 (28.4)	
	Moderate (10-14)	567 (12.4)	
	Moderately severe (15-19)	252 (5.5)	
	Severe (20-27)	105 (2.3)	
**Frequency of social media use for health information in the past week**			
	Never	338 (7.4)	
	Seldom	845 (18.4)	
	Sometimes	1603 (35)	
	Always	1794 (39.2)	
**Perceived susceptibility (score ≥3)**			
	Likelihood of contracting COVID-19 themselves	3104 (67.7)	
	Likelihood of family members contracting COVID-19	2887 (63.1)	
**Perceived severity (score ≥3)**			
	“COVID-19 will be transmitted from mother to child”	3630 (79.2)	
	“Maternal infection of COVID-19 will be more difficult to cure than the general population”	3827 (83.5)	
	“Maternal infection of COVID-19 will affect the health of the child”	3959 (86.4)	
**Worry (score ≥3)**			
	Worry that you will be infected with COVID-19 when you attend the prenatal check-up	3462 (75.5)	
	Worry that your hospital delivery arrangement will be infected due to COVID-19	3136 (68.5)	
	Worry that accompany delivery will not be available due to COVID-19	3197 (69.8)	
	Worry that child health services will be affected after delivery due to COVID-19	3391 (74)	

^a^PHQ-9: Patient Health Questionnaire-9.

### Correlation Between Study Variables

Among the sociodemographic and pregnancy-related characteristics, age had a negative correlation with depression (*r*=–0.03, *P*<.05). The frequency of using social media for health information had a positive correlation with perceived susceptibility (*r*=0.05, *P*<.001), perceived severity (*r*=0.11, *P*<.001), and worry due to COVID-19 (*r*=0.09, *P*<.001), but it had no significant correlation with depression. Perceived susceptibility (*r*=0.15, *P*<.001), perceived severity (*r*=0.19, *P*<.001), and worry due to COVID-19 (*r*=0.22, *P*<.001) all had a significant correlation with depression (Table 2).

**Table 2:** Correlation between study variables.

		Age	Education level	Parity	Gestational age	Complications^a^	Frequency of use^b^	Susceptibility^c^	Severity^d^	Worry^e^	Depression
**Age**											
	*r*	1	0.12	0.37	–0.02	0.06	0.04	–0.03	0.003	–0.02	–0.03
	*P* value	—^f^	<.001	<.001	.12	<.001	.01	.04	.85	.19	.03
**Education level**											
	*r*	0.12	1	–0.30	–0.10	0.05	0.25	0.01	–0.01	–0.05	–0.01
	*P* value	<.001	—	<.001	<.001	.001	<.001	.44	.41	<.001	.34
**Parity**											
	*r*	0.37	–0.30	1	0.09	–0.02	–0.08	–0.03	0.02	0.04	–0.004
	*P* value	<.001	<.001	—	<.001	.12	<.001	.08	.20	.01	.76
**Gestational age**											
	*r*	–0.02	–0.10	0.09	1	0.14	0.03	0.06	0.14	0.18	0.001
	*P* value	.12	<.001	<.001	—	<.001	.02	<.001	<.001	<.001	.92
**Complications^a^**											
	*r*	0.06	0.05	–0.02	0.14	1	0.02	0.01	0.03	0.03	0.001
	*P* value	<.001	.001	.12	<.001	—	.16	.40	.03	.02	.996
**Frequency of use^b^**											
	*r*	0.04	0.25	–0.08	0.03	0.02	1	0.05	0.11	0.09	–0.001
	*P* value	.01	<.001	<.001	.02	.16	—	.001	<.001	<.001	.955
**Susceptibility^c^**											
	*r*	–0.03	0.01	–0.03	0.06	0.01	0.05	1	0.31	0.37	0.15
	*P* value	.04	.44	.08	<.001	.40	.001	—	<.001	<.001	<.001
**Severity^d^**											
	*r*	0.003	–0.01	0.02	0.14	0.03	0.11	0.31	1	0.71	0.19
	*P* value	.85	.41	.20	<.001	.03	<.001	<.001	—	<.001	<.001
**Worry^e^**											
	*R*	–0.02	–0.05	0.04	0.18	0.03	0.09	0.37	0.71	1	0.22
	*P* value	.19	<.001	.01	<.001	.02	<.001	<.001	<.001	—	<.001
**Depression**											
	*r*	–0.03	–0.01	–0.004	0.001	0.001	–0.001	0.15	0.19	0.22	1
	*P* value	.03	.34	.76	.92	.996	.955	<.001	<.001	<.001	—

^a^Pregnancy-related complications.

^b^Frequency of social media use for health information.

^c^Perceived susceptibility of COVID-19.

^d^Perceived severity of COVID-19.

^e^Worry due to COVID-19.

^f^Not applicable.

### SEM Results

**Figure 1:**
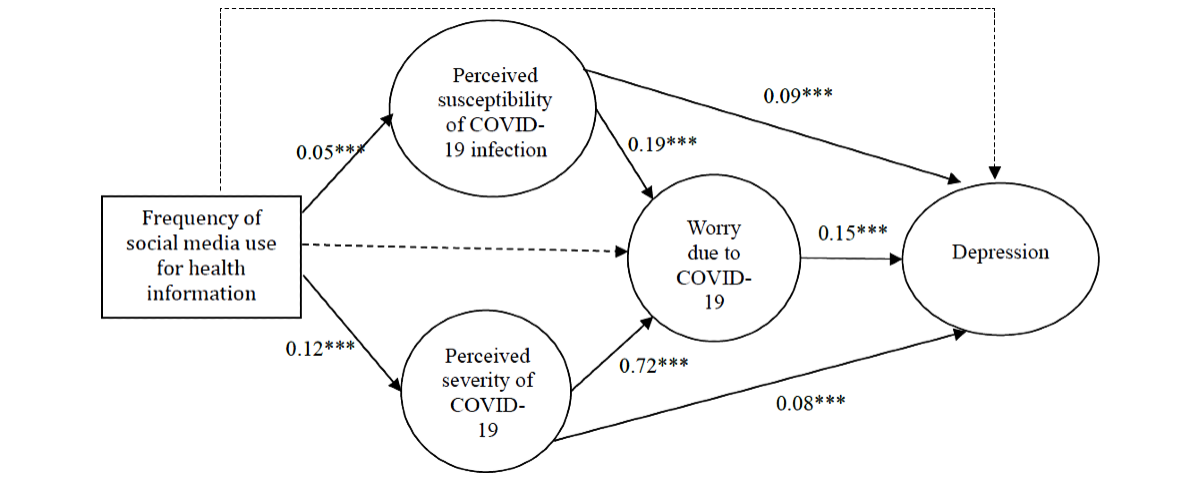
Structural equation model for social media use for health information, risk perceptions of COVID-19, worry due to COVID-19, and depression among pregnant women. The standardized coefficients of structural paths are shown after controlling significant background variables. Nonsignificant path is shown as a dotted line. Factor loadings and measurement errors have been omitted for clarity. ***P<.001.

Results from the confirmatory factor analysis suggested that the measurement model showed good fit to the data (*χ^2^*_48_=695.76; *P*=.01; CFI=0.99; IFI=0.99; RMSEA=0.05). All factor loadings were significant at *P*<.001 (Table 3). SEM results also showed that the structural model fitted the data well (*χ^2^*_57_=1143.3; *P<*.001; CFI=0.98; IFI=0.97; RMSEA=0.06). Frequency of social media use for health information was positively associated with perceived susceptibility (*β*=.05; *P*<.001) and perceived severity of COVID-19 (*β*=.12; *P*<.001), which in turn were positively associated with worry due to COVID-19 (*β*=.19 and *β*=.72, respectively; *P*<.001). Perceived susceptibility (*β*=.09; *P*<.001), perceived severity (*β*=.08; *P*<.001), and worry due to COVID-19 (*β*=.15; *P*<.001) all had a significant positive association with depression. In contrast, frequency of social media use for health information did not have a significant association with worry due to COVID-19 and depression (Figure 1). Bootstrapping analysis showed that the indirect effects of frequency of social media use for health information on worry due to COVID-19 (*β*=.09, 95% CI 0.07-0.12) and depression (*β*=.05, 95% CI 0.02-0.07) were both statistically significant.

**Table 3:** Unstandardized and standardized loadings for the measurement model.

Parameter estimates			Unstandardized loading (SE)		Standardized loading
**Perceived susceptibility of COVID-19**					
	Item 1	1.00		0.95	
	Item 2	0.99 (0.03)		0.92	
**Perceived severity of COVID-19**					
	Item 1	1.00		0.86	
	Item 2	1.05 (0.01)		0.93	
	Item 3	1.02 (0.01)		0.91	
**Worry due to COVID-19**					
	Item 1	1.00		0.82	
	Item 2	1.16 (0.02)		0.90	
	Item 3	1.12 (0.02)		0.85	
	Item 4	1.11 (0.02)		0.88	
**Depression**					
	Parcel score 1	1.00		0.85	
	Parcel score 2	1.01 (0.01)		0.93	
	Parcel score 3	0.73 (0.01)		0.79	

## Discussion

### Principal Findings

With the proliferation and rapid development of internet technologies and social networking sites, social media has become an important source of health information. In this study, more than one-third (39.2%) of the participants reported that they always used social media for obtaining health information during the COVID-19 pandemic. These findings are consistent with previous reports documenting an extensive use of social media for health information following the COVID-19 outbreak [25,26]. With the practices of physical and social distancing, individuals have increasingly turned to social media for information related to safety precautions and news updates related to COVID-19. Understanding how social media use for health information may change the information-seeking behaviors and health of pregnant women would be particularly valuable and meaningful.

To our knowledge, this is the first study that examined the associations between social media use for health information and depressive symptoms among pregnant women during the COVID-19 pandemic. It is important to note that, in the present study, nearly half (48.6%) of the participants were classified as having mild to severe depression—a figure that was significantly higher than that reported in the general population of pregnant women (ie, 7.4% to 12.8%) [50]. Furthermore, our findings show that the frequency of social media use for health information was indirectly associated with higher levels of worry and depression. These findings are in line with previous reports of a substantial proportion of pregnant women being confused about the complex or incorrect information available on the internet and experiencing heightened anxiety [24], as well as reports that have documented a positive relation between social media use and spread of fear and panic related to COVID-19 [51]. This study was conducted during the early phase of the COVID-19 pandemic, when China was significantly impacted; hence, it may be possible that participants in the present study did not only search for factual information about the pandemic, but they might also be exposed to the sharing of negative views, hot debate and arguments, and exaggerated worries toward COVID-19 via social media [30]. The mental impact of social media use during the COVID-19 period thus requires additional public health attention.

It would be important to understand the underlying mechanism through which social media use is associated with mental health. It is intriguing that using social media for health information was found to be indirectly associated with depression through perceived susceptibility and perceived severity about COVID-19, suggesting that social media use can affect the formation of risk perception of a pandemic. These findings are consistent with previous studies, which have documented that exposure to news media about a disease, such as H1N1, is associated with the formation of risk perceptions of the disease [52,53]. Social media has served as a useful tool for obtaining instant and up-to-date information during the COVID-19 outbreak. Nevertheless, since anyone can post on social media, it may also facilitate the sharing of inaccurate or unfiltered information, or the sharing of negative views, including uncertainty, severity, or suspicions of the disease. It is likely that exposure to the symptoms or complications related to COVID-19 may increase one’s *perceived severity* of the disease, whereas exposure to the statistics about disease prevalence or mortality rates may increase one’s *perceived susceptibility* to the disease. In general, the focus on negative information on social media may increase individuals’ level of risk perceptions toward the pandemic.

When an individual faces a health threat, they generate not only cognitive appraisal regarding the level of disease risk but also affective and emotional responses. Our study findings show that perceived susceptibility and severity are directly and indirectly associated with depression as a result of worry due to COVID-19. These findings are supported by the Appraisal Theory, which advocated that appraising an event as highly relevant and influential to one’s well-being leads to an emotional and affective response [40,41]. These findings concurred with previous studies, which showed that negative emotions during the COVID-19 outbreak could be amplified by misinformation fueled with rumors about the severity of the disease [54]. The perception about increased susceptibility and disease severity may mislead the public and increase uncontrolled panic associated with COVID-19 [44,45,55,56]. Our findings are also consistent to the extant literature that individuals who are exposed to excessive information about the harmful effects of a health issue might experience higher levels of health-related anxiety [57,58].

### Study Implications

Findings from this study suggest that the mental health of pregnant women during the COVID-19 pandemic warrants special attention. Screening for mental health problems, continuous monitoring of mental health status, and provision of psychological support throughout the pregnancy during a pandemic are highly warranted. Furthermore, these findings have raised the potential impact of social media in shaping risk perceptions and negative mental response among pregnant women. As social media has become one of the most important sources of health information during the COVID-19 pandemic, there is an urgent need to formulate strategies to minimize the potential negative effect that its use may have for pregnant women. It is suggested that accurate information and effective communication can be valuable to reduce misperception of risk, fear, and negative reactions toward the pandemic. It is important that appropriate social media strategies are developed to counter misinformation or negative information, and to ensure the credibility and accuracy of information shared during this period. Interventions to detect and counter inaccurate information about the media would also be important to reduce its negative impact.

Findings of our study also call for the need for intervention to guide pregnant women regarding the proper use of social media for health information. Alarming evidence suggests that most pregnant women perceive health information available on the internet to be reliable and that they rarely discuss the information with their physicians or midwives [18,20]. Without proper guidance, using social media for health information may lead to harmful consequences, such as information overload, or consumption of unreliable or misleading information [16,59,60]. Interventions are thus needed to empower pregnant women with the skills to identify credible source for obtaining health information, to provide thoughtful consideration of the veracity and quality of health information, and to process the information in an objective manner. Previous studies have also shown that people are likely to absorb negative information and react emotionally on social media. It is important to educate them about the potential bias that may occur in social media, and how these will affect their mental health during the pandemic. They should also be guided to manage their negative emotions, which may be elicited by exposure to stressful information and how to seek social support when they encounter stress as a result of social media use.

### Limitations

This study is subjected to several limitations. First, this study was cross-sectional in nature, so causality between the variables cannot be assumed. Nevertheless, it is important to note that the hypothesized association between social media use for health information, perceived susceptibility, perceived severity, worry due to COVID-19, and depression made theoretical sense. The cross-sectional nature of the study also precluded the opportunity to investigate change in the study variables. Second, only pregnant women from several provinces of China were recruited in this study; hence, the sample may not be generalizable to the whole population of pregnant women in China. Third, since no validated measures for measuring social media use for health information, perceived susceptibility, perceived severity, and worry related to COVID-19 were available, items were self-developed with reference to previous studies on other pandemics. The validity of survey items should therefore be cautioned. Fourth, as no information about those who did not participate in the study was available, no comparison between respondents and nonrespondents could be made. Finally, as the current model was based on the Appraisal Theory that highlights the important role of cognitive appraisal and resulting emotions, only cognitive and emotional factors were included in the study; other factors of depression, such as media literacy, resilience, confidence in fighting against the pandemic, and social support, have not been considered. Future studies could include a broader range of factors from different perspectives to allow a better understanding on the role of social media on mental health among pregnant women.

### Conclusions

Despite the limitations, given the scarcity of studies on the role of social media use for health information and mental health among pregnant women during the COVID-19 pandemic and the limited application of theoretical frameworks in understanding the topic, we believe that the findings of this study would provide valuable insights into the potential mental impact of social media use on mental health of pregnant women. This study shows that more than one-third of pregnant women surveyed reported that they always used social media for obtaining health information during the COVID-19 pandemic. Using social media for health information was indirectly associated with depression, based on our analyses of perceived susceptibility, perceived severity, and worry due to COVID-19. With the growing popularity of social media as a source of health information, interventions are needed to equip pregnant women with the skills to properly identify and access useful information from social media, as well as to educate them about the potential negative impact that social media use may pose to their health.

## Abbreviations

CFI: comparative fit index
IFI: incremental fit index
MERS: Middle East respiratory syndrome
QR: quick response
RMSEA: root mean square error of approximation
SARS: severe acute respiratory syndrome
SARS-CoV-2: severe acute respiratory syndrome coronavirus 2
SEM: structural equation modeling

## References

[1] Zhu N, Zhang D, Wang W, Li X, Yang B, Song J, Zhao X, Huang B, Shi W, Lu R, Niu P, Zhan F, Ma X, Wang D, Xu W, Wu G, Gao GF, Tan W (2020). A novel coronavirus from patients with pneumonia in China, 2019. N Engl J Med.

[2] World Health Organization COVID-19 Weekly Epidemiological Update.

[3] Jamieson DJ, Honein MA, Rasmussen SA, Williams JL, Swerdlow DL, Biggerstaff MS, Lindstrom S, Louie JK, Christ CM, Bohm SR, Fonseca VP, Ritger KA, Kuhles DJ, Eggers P, Bruce H, Davidson HA, Lutterloh E, Harris ML, Burke C, Cocoros N, Finelli L, MacFarlane KF, Shu B, Olsen SJ (2009). H1N1 2009 influenza virus infection during pregnancy in the USA. The Lancet.

[4] Yang H, Wang C, Poon LC (2020). Novel coronavirus infection and pregnancy. Ultrasound Obstet Gynecol.

[5] Castro P, Matos AP, Werner H, Lopes FP, Tonni G, Araujo Júnior E (2020). Covid-19 and pregnancy: an overview. Rev Bras Ginecol Obstet.

[6] Brooks SK, Webster RK, Smith LE, Woodland L, Wessely S, Greenberg N, Rubin GJ (2020). The psychological impact of quarantine and how to reduce it: rapid review of the evidence. The Lancet.

[7] Saulnier DD, Brolin K (2015). A systematic review of the health effects of prenatal exposure to disaster. Int J Public Health.

[8] Braunack-Mayer A, Tooher R, Collins JE, Street JM, Marshall H (2013). Understanding the school community's response to school closures during the H1N1 2009 influenza pandemic. BMC Public Health.

[9] Poon L, Yang H, Kapur A, Melamed N, Dao B, Divakar H, McIntyre H David, Kihara Anne B, Ayres-de-Campos Diogo, Ferrazzi Enrico M, Di Renzo Gian Carlo, Hod Moshe (2020). Global interim guidance on coronavirus disease 2019 (COVID-19) during pregnancy and puerperium from FIGO and allied partners: Information for healthcare professionals. Int J Gynaecol Obstet.

[10] Monk C, Lugo-Candelas C, Trumpff C (2019). Prenatal developmental origins of future psychopathology: mechanisms and pathways. Annu Rev Clin Psychol.

[11] Lee DT, Sahota D, Leung TN, Yip AS, Lee FF, Chung TK (2006). Psychological responses of pregnant women to an infectious outbreak: a case-control study of the 2003 SARS outbreak in Hong Kong. J Psychosom Res.

[12] Ng J, Sham A, Tang PL, Fung S (2004). SARS: pregnant women's fears and perceptions. Br J Midwifery.

[13] Davenport MH, Meyer S, Meah VL, Strynadka MC, Khurana R (2020). Moms are not OK: COVID-19 and maternal mental health. Front Glob Womens Health.

[14] Hessami K, Romanelli C, Chiurazzi M, Cozzolino M (2020). COVID-19 pandemic and maternal mental health: a systematic review and meta-analysis. J Matern Fetal Neonatal Med.

[15] Durankuş F, Aksu E (2020). Effects of the COVID-19 pandemic on anxiety and depressive symptoms in pregnant women: a preliminary study. J Matern Fetal Neonatal Med.

[16] Rice RE (2006). Influences, usage, and outcomes of Internet health information searching: multivariate results from the Pew surveys. Int J Med Inform.

[17] Siliquini R, Ceruti M, Lovato E, Bert F, Bruno S, De Vito E, Liguori G, Manzoli L, Messina G, Minniti D, La Torre G (2011). Surfing the internet for health information: an italian survey on use and population choices. BMC Med Inform Decis Mak.

[18] Gao L, Larsson M, Luo S (2013). Internet use by Chinese women seeking pregnancy-related information. Midwifery.

[19] Javanmardi M, Noroozi M, Mostafavi F, Ashrafi-Rizi H (2018). Internet usage among pregnant women for seeking health information: a review article. Iran J Nurs Midwifery Res.

[20] Larsson M (2009). A descriptive study of the use of the internet by women seeking pregnancy-related information. Midwifery.

[21] Lagan B, Sinclair M, Kernohan W (2010). Internet use in pregnancy informs women's decision making: a web-based survey. Birth.

[22] Bert F, Gualano MR, Brusaferro S, De Vito E, de Waure C, La Torre G, Manzoli L, Messina G, Todros T, Torregrossa MV, Siliquini R (2013). Pregnancy e-health: a multicenter Italian cross-sectional study on Internet use and decision-making among pregnant women. J Epidemiol Community Health.

[23] Sayakhot P, Carolan-Olah M (2016). Internet use by pregnant women seeking pregnancy-related information: a systematic review. BMC Pregnancy Childbirth.

[24] De Santis M, De Luca C, Quattrocchi T, Visconti D, Cesari E, Mappa I, Nobili E, Spagnuolo T, Caruso A (2010). Use of the internet by women seeking information about potentially teratogenic agents. Eur J Obstet Gynecol Reprod Biol.

[25] Pan Y, Xin M, Zhang C, Dong W, Fang Y, Wu W, Li M, Pang J, Zheng Z, Wang Z, Yuan J, He Y (2020). Associations of mental health and personal preventive measure compliance with exposure to COVID-19 information during work resumption following the COVID-19 outbreak in China: cross-sectional survey study. J Med Internet Res.

[26] Cuello-Garcia C, Pérez-Gaxiola Giordano, van Amelsvoort L (2020). Social media can have an impact on how we manage and investigate the COVID-19 pandemic. J Clin Epidemiol.

[27] Zarocostas J (2020). How to fight an infodemic. The Lancet.

[28] Seo M (2019). Amplifying Panic and Facilitating Prevention: Multifaceted Effects of Traditional and Social Media Use During the 2015 MERS Crisis in South Korea. Journalism & Mass Communication Quarterly.

[29] Choi D, Yoo W, Noh G, Park K (2017). The impact of social media on risk perceptions during the MERS outbreak in South Korea. Comput Human Behav.

[30] Liao Q, Yuan J, Dong M, Yang L, Fielding R, Lam WWT (2020). Public engagement and government responsiveness in the communications about COVID-19 during the early epidemic stage in China: infodemiology study on social media data. J Med Internet Res.

[31] Gao J, Zheng P, Jia Y, Chen H, Mao Y, Chen S, Wang Y, Fu H, Dai J (2020). Mental health problems and social media exposure during COVID-19 outbreak. PLoS One.

[32] Riello M, Purgato M, Bove C, MacTaggart D, Rusconi E (2020). Prevalence of post-traumatic symptomatology and anxiety among residential nursing and care home workers following the first COVID-19 outbreak in Northern Italy. R Soc Open Sci.

[33] Hadjistavropoulos HD, Craig KD, Hadjistavropoulos T (1998). Cognitive and behavioral responses to illness information: the role of health anxiety. Behav Res Ther.

[34] Garfin D, Silver R, Holman E (2020). The novel coronavirus (COVID-2019) outbreak: amplification of public health consequences by media exposure. Health Psychol.

[35] Liu X, Lo V (2014). Media exposure, perceived personal impact, and third-person effect. Media Psychology.

[36] Determann D, de Bekker-Grob EW, French J, Voeten HA, Richardus JH, Das E, Korfage IJ (2016). Future pandemics and vaccination: public opinion and attitudes across three European countries. Vaccine.

[37] Carpenter CJ (2010). A meta-analysis of the effectiveness of health belief model variables in predicting behavior. Health Commun.

[38] Sengupta S, Wang H (2014). Information sources and adoption of vaccine during pandemics. Int J Pharm Healthc Mark.

[39] Pérez-Fuentes MDC, Molero Jurado MDM, Martos Martínez Á, Gázquez Linares JJ (2020). Threat of COVID-19 and emotional state during quarantine: positive and negative affect as mediators in a cross-sectional study of the Spanish population. PLoS One.

[40] Smith CA, Ellsworth PC (1985). Patterns of cognitive appraisal in emotion. J Pers Soc Psychol.

[41] Smith CA, Kirby LD (2009). Putting appraisal in context: toward a relational model of appraisal and emotion. Cognition & Emotion.

[42] Ding Y, Xu J, Huang S, Li P, Lu C, Xie S (2020). Risk perception and depression in public health crises: evidence from the COVID-19 crisis in China. Int J Environ Res Public Health.

[43] Han Q, Zheng B, Agostini M, Bélanger JJ, Gützkow B, Kreienkamp J, Reitsema AM, van Breen JA, Collaboration P, Leander NP (2021). Associations of risk perception of COVID-19 with emotion and mental health during the pandemic. J Affect Disord.

[44] Tran BX, Nguyen HT, Le HT, Latkin CA, Pham HQ, Vu LG, Le XTT, Nguyen TT, Pham QT, Ta NTK, Nguyen QT, Ho CSH, Ho RCM (2020). Impact of COVID-19 on economic well-being and quality of life of the Vietnamese during the national social distancing. Front Psychol.

[45] Tee ML, Tee CA, Anlacan JP, Aligam KJG, Reyes PWC, Kuruchittham V, Ho RC (2020). Psychological impact of COVID-19 pandemic in the Philippines. J Affect Disord.

[46] Bian C, Li C, Duan Q, Wu H (2011). Reliability and validity of patient health questionnaire: depressive syndrome module for outpatients. Scientific Research and Essays.

[47] Zhang Y, Ting R, Lam M, Lam J, Nan H, Yeung R, Yang W, Ji L, Weng J, Wing Y, Sartorius N, Chan JC (2013). Measuring depressive symptoms using the Patient Health Questionnaire-9 in Hong Kong Chinese subjects with type 2 diabetes. J Affect Disord.

[48] Li Z, Dai J, Wu N, Gao J, Fu H (2019). The mental health and depression of rural-to-urban migrant workers compared to non-migrant workers in Shanghai: a cross-sectional study. Int Health.

[49] Kline R (2005). Principles and practice of structural equation modeling (2nd edition).

[50] Bennett HA, Einarson A, Taddio A, Koren G, Einarson TR (2004). Prevalence of depression during pregnancy: systematic review. Obstetrics & Gynecology.

[51] Ahmad AR, Murad HR (2020). The impact of social media on panic during the COVID-19 pandemic in Iraqi Kurdistan: online questionnaire study. J Med Internet Res.

[52] Chang C (2012). News coverage of health-related issues and its impacts on perceptions: Taiwan as an example. Health Commun.

[53] Chung JE (2016). A smoking cessation campaign on Twitter: understanding the use of Twitter and identifying major players in a health campaign. J Health Commun.

[54] Islam M, Sarkar T, Khan S, Mostofa Kamal A-H, Hasan S, Kabir A, Yeasmin D, Islam M, Amin Chowdhury Kamal Ibne, Anwar K, Chughtai A, Seale H (2020). COVID-19-related infodemic and its impact on public health: a global social media analysis. Am J Trop Med Hyg.

[55] Ho CS, Chee CY, Ho RC (2020). Mental health strategies to combat the psychological impact of coronavirus disease 2019 (COVID-19) beyond paranoia and panic. Ann Acad Med Singap.

[56] Cuan-Baltazar JY, Muñoz-Perez MJ, Robledo-Vega C, Pérez-Zepeda MF, Soto-Vega E (2020). Misinformation of COVID-19 on the internet: infodemiology study. JMIR Public Health Surveill.

[57] Blackburn J, Fischerauer SF, Talaei-Khoei M, Chen NC, Oh LS, Vranceanu A (2019). What are the implications of excessive internet searches for medical information by orthopaedic patients?. Clin Orthop Relat Res.

[58] Norr AM, Capron DW, Schmidt NB (2014). Medical information seeking: impact on risk for anxiety psychopathology. J Behav Ther Exp Psychiatry.

[59] Skinner H, Biscope S, Poland B, Goldberg E (2003). How adolescents use technology for health information: implications for health professionals from focus group studies. J Med Internet Res.

[60] Cline R, Haynes K (2001). Consumer health information seeking on the internet: the state of the art. Health Educ Res.

